# Development of an Oral Liquid Formulation of Nicardipine Hydrochloride Compounded with Simple Excipients for the Treatment of Pediatric Hypertension

**DOI:** 10.3390/pharmaceutics15020446

**Published:** 2023-01-29

**Authors:** Marine Cavelier, Henri Gondé, Damien Costa, Fabien Lamoureux, Tony Pereira, Nimrod Buchbinder, Rémi Varin, Charles Hervouët

**Affiliations:** 1 CHU Rouen, Department of Pharmacy, F-76000 Rouen, France; 2 CHU Rouen, Department of Pharmacy, Normandie University, UNIROUEN, U1234, F-76000 Rouen, France; 3 CHU Rouen, Department of Parasitology-Mycology, Normandie University, UNIROUEN, EA7510 ESCAPE, F-76000 Rouen, France; 4 CHU Rouen, Department of Pharmacology, F-76000 Rouen, France; 5 CHU Rouen, Department of Pediatric Oncology and Hematology, F-76000 Rouen, France

**Keywords:** nicardipine hydrochloride, oral solution, pediatric formulation, compounding, stability, hypertension

## Abstract

Nicardipine hydrochloride is an anti-hypertensive drug that is used off-label to treat hypertension in children. A previous oral formulation of nicardipine hydrochloride was developed using a commercial vehicle as an excipient. However, ready-to-use vehicles are prone to supply shortages, and their composition may undergo substantial modifications. The aim of this study was to propose a new oral formulation of nicardipine hydrochloride 2 mg/mL using simple excipients. The formulation included hydroxypropylmethylcellulose, simple syrup, polysorbate 80, sodium saccharin, citrate buffer, strawberry flavor and 0.2% potassium sorbate. The uniformity of content was maintained before and after agitation. Nicardipine hydrochloride concentration assessed by HPLC-MS/MS remained above 90% for 365 days before opening and for 28 days after opening. pH and osmolality were maintained throughout the study, and no microbial contamination was observed. The uniformity of mass of the delivered doses was evaluated using four different devices. A new oral formulation of nicardipine hydrochloride 2 mg/mL was developed using simple and safe excipients. Pharmacological and clinical parameters remain to be assessed and compared with those of the previous formulation.

## 1. Introduction

Prescribers in pediatrics are commonly faced with the absence of medicines with dosages and/or pharmaceutical forms suitable for children [1,2]. As a result, prescribers often need hospital and community pharmacies for extemporaneous compounding of drugs in either capsule or liquid forms. Oral liquid forms can be administered in children from birth, allowing dosing flexibility and facilitating administration.

Hypertension affects 2 to 4% of children and in most cases is a consequence of a primary condition [3,4]. To date, few anti-hypertensive medicines are available in pediatrics, and the pharmaceutical compounding of angiotensin-converting enzyme inhibitors (e.g., captopril) and calcium-channel blockers (e.g., amlodipine and nicardipine hydrochloride) is common [5].

**Figure 1:**
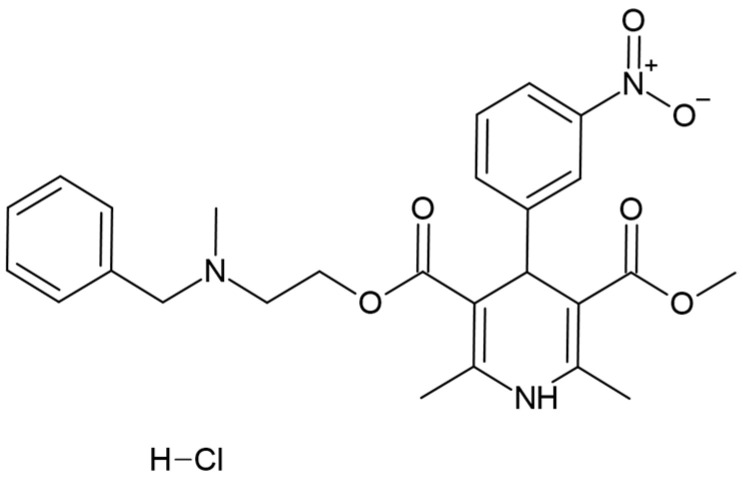
Chemical structure of nicardipine hydrochloride.

Nicardipine hydrochloride (Figure 1) is a Biopharmaceutical Classification System (BCS) class II molecule, i.e., with high gastrointestinal tract permeability but poor solubility in water limiting its bioavailability [6]. Nicardipine hydrochloride solubility in phosphate buffer and citrate buffer did not exceed 5–6 mg/mL but was enhanced in acetate, propionate and butyrate buffers [7].

The formulation of a class II drug for oral administration is often challenging, including crystalline solid formulations, lipid formulations and amorphous formulations [8].

The short half-life of nicardipine hydrochloride is of interest in a hospital setting, allowing rapid dose adjustment. Currently, only 20 mg tablets and extended-release 50 mg capsules are available, but these are not suitable in pediatrics since the usual dose of nicardipine hydrochloride ranges from 0.25 to 3 mg/kg/day, administered in two or three individual doses [9].

The selection of excipients with suitable safety and tolerability along with appropriate physicochemical properties is of paramount importance in pediatric formulation development. Some excipients such as propylene glycol and ethanol cannot be metabolized because of the immaturity of organs leading to adverse effects. In addition, taste masking which often relies on sweeteners is important to improve palatability. Texture and viscosity are also key attributes for patient acceptability [10,11,12,13,14].

A previous oral formulation of nicardipine hydrochloride 2 mg/mL was reported, using a commercial vehicle as an excipient [15]. Complex vehicles allow easy compounding of oral liquid formulations but may be affected by supply shortages, batch withdrawal and modifications in their composition. These products may contain additives such as thickening, buffering, sweetening and flavoring agents. Parabens, sodium benzoate or potassium sorbate are also included as preservatives in most commercial vehicles. Although substances in the composition of commercial vehicles are often listed, the relative quantity of each compound is never described.

The objective of this study was to develop a new oral liquid formulation of nicardipine hydrochloride with simple excipients. A pre-formulation study was first conducted. The formulation was then characterized, and physicochemical and microbiological stability was assessed.

## 2. Materials and Methods

### 2.1. Materials

Nicardipine hydrochloride, polysorbate 80, hydroxypropylmethylcellulose (HPMC) 4000, sorbitol, sodium saccharin and raspberry and strawberry flavors were purchased from Inresa (Bartenheim, France). Citric acid monohydrate, sodium citrate, potassium sorbate, sucrose, glycerol and carboxymethylcellulose were purchased from Cooper (Melun, France) and sterile water (Versylene^®^) from Fresenius Kabi (Bad Homburg vor der Höhe, Germany).

Reagents were of analytical grade and included acetonitrile, ammonium formate, formic acid, water, methanol, ethanol and sodium hydroxide, purchased from Carlo Erba (Val de Reuil, France). Hydrochloric acid was obtained from VWR Chemicals (Leuven, Belgium) and hydrogen peroxide from Gifrer (Decines Charpieu, France). Nicardipine hydrochloride -d3 was purchased from Toronto Research Chemicals (Toronto, ON, Canada).

Microorganisms (*Pseudomonas aeruginosa* (ATCC 9027), *Staphylococcus aureus* (ATCC 6538), *Escherichia coli* (ATCC 8739), *Bacillus subtilis* (ATCC 6633), *Candida albicans* (ATCC 10231) and *Aspergillus brasiliensis* (ATCC 16404)) were purchased from Bioreference laboratory (Douai, France).

### 2.2. Analytical Method

#### 2.2.1. Chromatographic Conditions

Nicardipine hydrochloride was quantified according to previously validated high-performance liquid chromatography–tandem mass spectrometry (HPLC-MS/MS) method using a Shimadzu prominence HPLC system (Shimadzu^®^, Kyoto, Japan) coupled to a 3200 QTRAP triple quadrupole mass spectrometer (Sciex^®^, Framingham, MA, USA) and an Alltima HP C18 HL 3 μm 150 × 3 mm column (Avantor^®^, VWR^®^, Rosny-sous-Bois, France) [15].

The mobile phase consisted of acetonitrile and 2 mM ammonium formate with 0.2% formic acid in water (70/30, *v*/*v*).

Data acquisition and analysis were performed using Analyst^®^ 1.6.3 software (Sciex, Framingham, MA, USA).

#### 2.2.2. Sample Preparation

Nicardipine hydrochloride 2 mg/mL solution was obtained by solvating 20 mg of nicardipine hydrochloride powder in 10 mL of 50:50 *v*/*v* water–methanol mixture. One milliliter of 2 mg/mL solution was diluted with 1 mL ethanol. Fifty microliters of this solution was subsequently diluted with 10 mL of 50:50 *v*/*v* water–methanol mixture in volumetric flask to reach 5 μg/mL. Finally, a 5 µg/mL solution was diluted in a 20 mL volumetric flask with 50:50 water–methanol mixture to obtain calibrators (85, 92.5, 100, 107.5 and 115 ng/mL) and validation standards (85, 100 and 115 ng/mL). All solutions were freshly prepared on each day of analysis. Samples from nicardipine hydrochloride oral formulation for determination of the uniformity of content and for the stability study were prepared following the same protocol.

Internal standards were prepared by solvating 1 mg of nicardipine-d3 hydrochloride powder with 1 mL of methanol, followed by dilution in 10 mL of methanol in volumetric flask to reach 40 μg/mL. Internal standards were added to calibrators, validation standards and samples to reach 100 ng/mL.

### 2.3. Retrospective Analysis of Patients Treated with Nicardipine 2 mg/mL Oral Formulation

Children who were treated with the previous nicardipine hydrochloride 2 mg/mL oral formulation in our center from 1 January 2020 to 31 December 2020 were identified using an internal compounding software program. Electronic medical files (CDP2^®^ software, C.PAGE patient files) and prescriptions were consulted to collect demographic data and the prescribed doses of nicardipine. The study obtained ethics approval provided by the institutional ethics committee (CERDE-HLJ: Comité d’Ethique pour la Recherche sur Données Existantes et/ou Hors Loi Jardé) with the number E2023-02.

### 2.4. Pre-Formulation

#### 2.4.1. Excipients

The excipients used to improve visual appearance were carboxymethycellulose, hydroxypropylmethycellulose, citric acid, sodium citrate and polysorbate 80. The excipients used to improve palatability were simple syrup, sorbitol, sodium saccharin and strawberry and raspberry flavors.

#### 2.4.2. Efficacy of Antimicrobial Preservation

Strains (*S. aureus*, *P. aeruginosa*, *E. coli*, *C. albicans* and *A. brasiliensis*) were inoculated on appropriate agar plate and incubated at 30 °C for fungi and 35 °C for bacteria. Cultures were harvested and dispersed in 0.85% sodium chloride until the desired optical density was reached using a densitometer (Densimat, Biomerieux, Marcy-l’étoile, France). For *A. brasiliensis*, polysorbate 80 was added to disperse spores. One hundred microliters of the inoculum was transferred into bottles containing 10 mL of nicardipine hydrochloride 2 mg/mL with 0.2% or 0.3% potassium sorbate to reach a concentration of 10^5^ to 10^6^ microorganisms/mL. Bottles were stored at room temperature and protected from light. Bacteria and fungi were counted on days 0, 14 and 28 after plating on duplicate agars by surface spread. The test was compliant with European Pharmacopoeia (Eur.Ph.) (monograph 5.1.3) if a logarithmic reduction (>3log_10_ for bacteria and >1log_10_ for fungi) of the initial inoculum was observed on day 14 with no growth between day 14 and day 28.

### 2.5. Characterization of the Final Formulation

#### 2.5.1. Light Microscopy

Nicardipine hydrochloride 2 mg/mL was prepared according to the standard operating procedure (Section 3.2.3). For the preparation of matrix without nicardipine hydrochloride 2 mg/mL, the same standard operating procedure was applied, except that nicardipine hydrochloride was not added to the mixture. Negative control was water (Versylene^®^), and positive control was a commercial suspension of paracetamol (Doliprane^®^ 2.4%, Sanofi-Aventis, Gentilly, France). Ten microliters of each compound was sampled and dropped in a Glasstic^®^ Slide 10 with Grids (Kova International Inc., Garden Grove, CA, USA). Visualization was performed under a light microscope (Eclipse C-TEP3, Nikon, Tokyo, Japan) using 10X magnification.

#### 2.5.2. Uniformity of Content

Two milliliters was sampled from the top, middle and bottom of 50 mL bottles from three different batches. The test was performed before and after agitation (10 successive inversions). Samples were analyzed by HPLC-MS/MS and compared using a Wilcoxon test.

### 2.6. Stability Study

#### 2.6.1. Chemical Stability

Three batches of nicardipine hydrochloride 2 mg/mL were packaged in 50 mL and 250 mL amber glass bottles. Bottles were stored at room temperature (25 ± 2 °C) and refrigerated temperature (5 ± 3 °C). Nicardipine hydrochloride concentration was monitored in 50 mL bottles on days 0, 60, 90, 183, 268 and 365. To evaluate the stability of “in-use” conditions, 250 mL bottles were opened three times a day to collect 1 mL of nicardipine hydrochloride 2 mg/mL using the same syringe throughout the study, and nicardipine hydrochloride concentration was measured on days 0, 7, 14 and 28. Each analysis was performed in triplicate.

#### 2.6.2. Physical Stability

Organoleptic appreciation (i.e., appearance, color and odor), pH and osmolality were evaluated in the three batches at days 0, 7, 14, 28, 60, 90, 183, 268 and 365. The osmolality was measured with an osmometer (Osmometer Löser type 16, Berlin, Germany) in triplicate and the pH using a pH meter (Inolab pH level 2P, WTW, Nuremberg, Germany). Equipment was calibrated before use.

#### 2.6.3. Microbiological Study

1. Method validation

Diluted nicardipine hydrochloride 2 mg/mL (1/10) was inoculated with 100 colony-forming units (CFU) of *S. aureus*, *P. aeruginosa*, *B. subtilis*, *C. albicans* and *A. brasiliensis* using pour-plate method. For the determination of total aerobic microbial count (TAMC), trypto-casein-soya agars were incubated at 35 °C for 3 days. Sabouraud agars for total combined yeast/mold count (TYMC) were incubated at 20–25 °C for 5 days. Positive controls were inoculated in tryptone salt broth. The test was compliant with Eur.Ph. (monograph 2.6.12) if the growth of microorganisms did not differ by more than a factor of 2 from the positive control.

Test for *E. coli* was performed according to Eur.Ph. (monograph 2.6.13). Briefly, 100 CFU were inoculated in diluted nicardipine hydrochloride. After 24 h incubation (35 °C) followed by incubation in MacConkey broth during 24 h (44 °C), MacConkey agars were inoculated and incubated for 3 days at 35 °C. The test was compliant if pink colonies were observed on MacConkey agars.

Each analysis was performed in duplicate.

2. Microbiological stability

The study was performed in bottles of 50 mL and 250 mL stored at 25 ± 2 °C or 5 ± 3 °C. For determination of microbial contamination “before opening”, bottles of 50 mL were opened (i.e., bottles were only opened for analysis) on days 0, 14, 35, 63, 91, 183, 268 and 365. Microbial contamination “after opening” was determined on days 0, 14 and 35 from samples of 250 mL bottles sampled three times a day using a syringe washed with water after sampling.

Specifications of Eur.Ph. (monograph 5.1.4) are the absence of *Escherichia coli* and a count of total aerobic bacteria equal to a maximum of 100 CFU/mL and that of mold/yeast to a maximum of 10 CFU/mL.

### 2.7. Uniformity and Accuracy of Delivered Doses from Multidose Bottles

According to Eur.Ph. (monograph 2.9.27), doses of 4 mL were sampled and weighed twenty times. The test was performed using the following devices: 5 mL Luer-lock syringes (Plastipak^®^, Becton Dickinson, Franklin Lakes, NJ, USA) with an adapter cap (Spruyt hillen, IJsselstein, Netherlands), 5 mL Luer syringes (Injekt^®^; BBraun, Melsungen, Germany), with an adapter cap (Spruyt hillen, IJsselstein, The Netherlands), and 5 mL syringes for oral use (Nutrifit^®^; Vygon, Compli, Italy) combined or not with a press-in bottle adapter (Medicina, Bolton, UK). The test was compliant if no more than two of the individual masses deviated from the average mass by more than 10% and none deviated by more than 20%.

### 2.8. Data Analysis

GraphPad Prism^®^ software version 8.3.0 (La Jolla, CA, USA) was used for graphs and statistical analysis. *p*-values of less than 0.05 were considered statistically significant.

## 3. Results

### 3.1. Retrospective Analysis of Patients Treated with Nicardipine Hydrochloride 2 mg/mL Oral Formulation

A previous oral formulation of nicardipine hydrochloride 2 mg/mL was reported using a commercial vehicle as an excipient [15]. The choice of the concentration was based on a retrospective analysis of the nicardipine hydrochloride capsules compounded in our center before the implementation of an oral liquid formulation. To determine whether this concentration of 2 mg/mL was still suitable, the characteristics of patients who were administered the previous nicardipine hydrochloride oral solution were reviewed (Table 1).

Median age was 4.5 years, and the median dose of nicardipine hydrochloride was 8.2 mg corresponding to a volume of 4.1 mL of the oral formulation of nicardipine hydrochloride 2 mg/mL. This was consistent with current guidelines. Thus, we concluded that a concentration of 2 mg/mL was still suitable to treat hypertension in children.

### 3.2. Pre-Formulation

#### 3.2.1. Selection of Excipients

The solubility of nicardipine hydrochloride in aqueous media is poor, and nicardipine hydrochloride displays a strong bitter taste. Thus, a formulation was developed with the following features immediately after compounding and after several days of storage: (i) homogeneous to the naked eye, (ii) good palatability and (iii) sufficient apparent viscosity to the naked eye (Table 2).

The first formulations included simple syrup (formulation F1) or a 50/50 mixture of simple syrup and water (F2), with the addition of 1% polysorbate 80. F1 was not suitable as the glucose content was too high (simple syrup contains 65% glucose), whereas F2 displayed a bitter taste.

Then, water formulations containing a thickening agent that was either CMC 0.1% and 0.5% (F3) or HPMC 0.5% (F4) and 0.25% (F5) were tested. F3 was opalescent with visible aggregates of nicardipine on the magnet bar. F4 and F5 were also opalescent, and nicardipine hydrochloride seemed to be dispersed to the naked eye. The apparent viscosity of F4 was too high, in contrast with F5 whose viscosity seemed to be suitable for oral use. However, F5 had a bitter taste.

Sorbitol (F6) or sodium saccharin (F7) were added to F5 as sweetening agents. The bitter taste of nicardipine hydrochloride was masked in F7 using 0.1% sodium saccharin. Sedimentation was observed in formulations F4 to F7 after several days, which could not be resuspended. The addition of 0.1% citrate buffer to decrease the pH from 5.4 to 4.8 slightly improved the stability of the formulation, but sedimentation was still observed after several days (F8). Thus, 1%, 0.75% or 0.5% polysorbate 80 were added (respectively, F9, F10 and F11). Only F9 and F10 were visually clear, but they displayed a strong unpleasant taste due to nicardipine and polysorbate despite the presence of 0.1% (F9 and F10) or 0.15% sodium saccharin (F12). The addition of flavoring agents did not totally mask the bitter taste (F13), whereas the co-addition of strawberry flavor and 38% simple syrup improved palatability (F14). Formulation F14 was considered visually clear and suitable for children. F14 was selected for subsequent studies.

#### 3.2.2. Efficacy of Antimicrobial Preservation

On day 0, 0.35 × 10^5^ to 6.9 × 10^5^ microorganisms/mL were counted in the formulation containing 0.2% or 0.3% potassium sorbate as an antimicrobial preservative. After 14 days of storage, no more microorganisms were detected in bottles containing 0.2% or 0.3% potassium sorbate, and no growth was observed on day 28 (Table 3) indicating compliance with Eur.Ph. for both conditions. It was therefore decided to add 0.2% potassium sorbate to the final formulation.

#### 3.2.3. Final Formulation

The composition of the nicardipine hydrochloride 2 mg/mL oral formulation is presented in Table 4.

The standard operating procedure first included the preparation of simple syrup, the dissolution of HPMC and the dissolution of nicardipine hydrochloride. Simple syrup 65% *w*/*w* was prepared by mixing 247 g of sucrose in water heated to 80–85 °C. This solution was filtered, and additional water was added to reach a final weight of 380 g. The dissolution of HPMC was obtained by mixing 2.5 g of HPMC and 150 g of water while stirring. Two grams of nicardipine hydrochloride was added to a mixture of sterile water (480 g) and polysorbate 80 (7.5 g), and the dissolution was obtained after 1 h of stirring away from light. The next step of the standard operating procedure was to mix all the components together. First, potassium sorbate, sodium saccharin, citric acid, sodium citrate and HPMC were added to the nicardipine hydrochloride solution previously obtained. Then, simple syrup and strawberry flavor were added while stirring. Finally, sterile water was added to reach a final concentration of 2 mg/mL. The nicardipine hydrochloride 2 mg/mL formulation was limpid and yellow in color. The preparation was packaged in amber type 3 glass bottles and closed with polypropylene caps.

### 3.3. Characterization of the Final Formulation

#### 3.3.1. Light Microscopy

**Figure 2:**
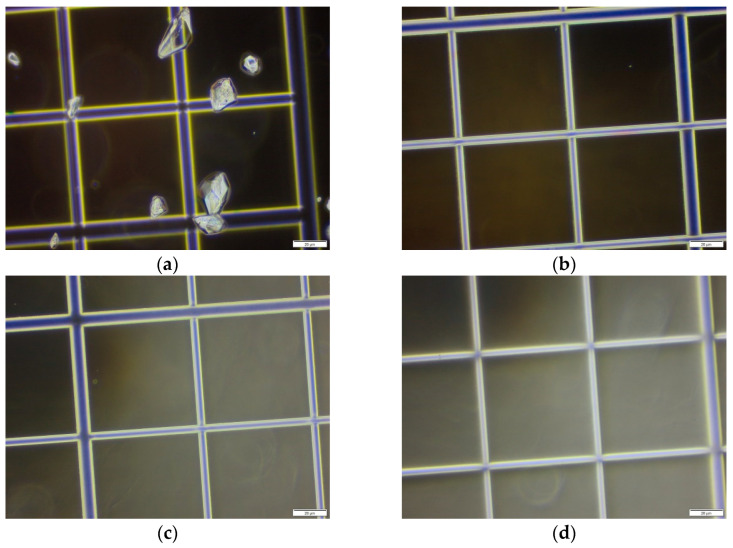
Observation under light microscope of (**a**) suspension of paracetamol (Doliprane^®^ 2.4%); (**b**) water; (**c**) matrix without nicardipine hydrochloride 2 mg/mL; (**d**) nicardipine hydrochloride 2 mg/mL formulation. Scale bar = 20 µm.

Using a commercial suspension of paracetamol for oral use as a positive control, crystals of approximately 10 to 30 µm in length were visualized under light microscopy (Figure 2a). In contrast, no crystals were observed in the water, the matrix without nicardipine hydrochloride and the nicardipine hydrochloride 2 mg/mL oral formulation (Figure 2b–d). This observation suggested that nicardipine hydrochloride was dissolved rather than dispersed in a suspension.

#### 3.3.2. Uniformity of Content

**Figure 3:**
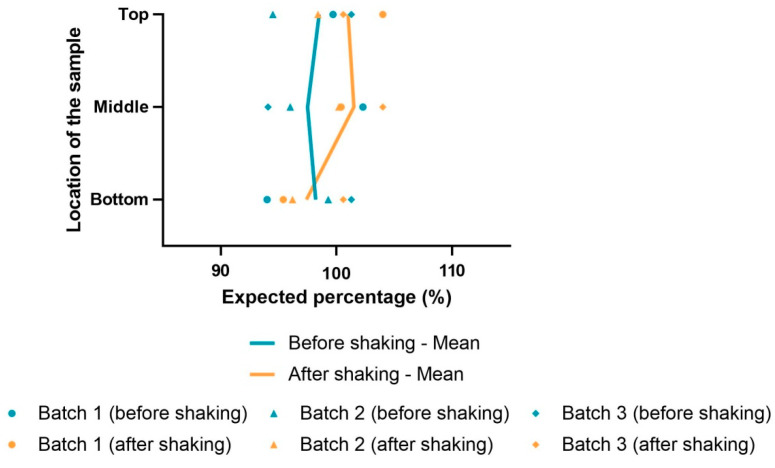
Uniformity of content of 50 mL bottles containing nicardipine hydrochloride 2 mg/mL formulation before and after agitation.

To explore the uniformity of nicardipine hydrochloride content in bottles, sampling was performed at the top, in the middle and at the bottom, and nicardipine hydrochloride concentration was determined by HPLC-MS/MS. Nicardipine hydrochloride concentration was 98.5%, 97.5% and 98.2% of the expected quantity before agitation and 101%, 101.5% and 97.4% after agitation, respectively, at the top, in the middle and at the bottom of the bottles (Figure 3). The amount of nicardipine hydrochloride was not different at the top (*p* = 0.5), in the middle (*p* = 0.5) and at the bottom (*p* = 0.75) of the bottles whether there was prior agitation or not. Therefore, agitation was not required prior to sampling to maintain uniform concentration in the bottles.

### 3.4. Stability Study

#### 3.4.1. Chemical Stability

1. Long-term storage conditions

**Figure 4:**
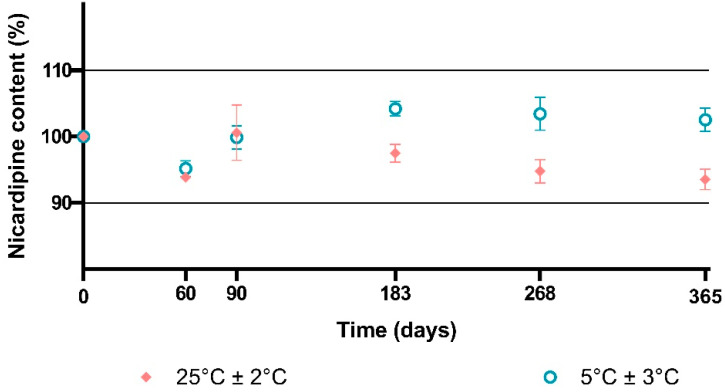
Chemical stability of nicardipine hydrochloride in compounded oral solution at 2 mg/mL for 365 days. Note: Results are expressed as the mean of the nine samples (three test samples for each of the three batches), and error bars are defined as SD.

Concentrations of nicardipine hydrochloride remained stable for 365 days in bottles stored at 5 °C (Figure 4). In contrast, a trend toward a slight decrease in nicardipine hydrochloride was observed from day 183 to day 365 in bottles stored at room temperature even if nicardipine hydrochloride remained above 90% of initial concentrations throughout the study.

2. In-use conditions

**Figure 5:**
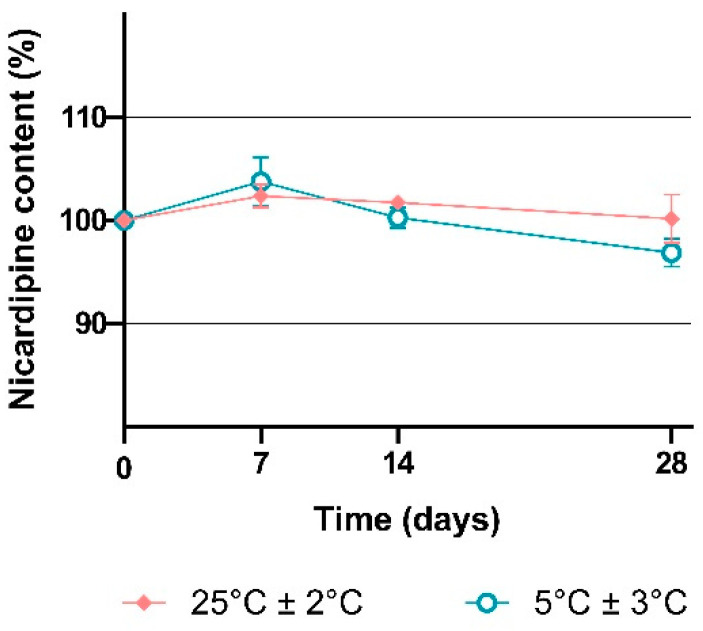
Chemical stability of nicardipine hydrochloride in compounded oral solution at 2 mg/mL for 28 days in “in-use conditions”. Note: Results are expressed as the mean of the nine samples (three test samples for each of the three batches), and error bars are defined as SD.

Nicardipine hydrochloride is administered b.i.d. or t.i.d., implying regular opening of the bottle and regular sampling. To evaluate the stability of nicardipine hydrochloride 2 mg/mL in these in-use conditions, we sampled bottles stored at 5 °C or 25 °C three times a day for 28 days. Nicardipine hydrochloride content remained within the 90–110% range for both storage conditions (Figure 5).

Vials were opened daily, and 1 mL of the solution was sampled three times a day.

#### 3.4.2. Physical Stability

The formulation from three different batches stored away from light, at room temperature (25 ± 2 °C) or in refrigerated conditions (5 ± 3 °C) for 365 days remained clear and yellow in color and retained the strawberry smell. pH and osmolality also remained constant throughout the study for both storage conditions (Table 5).

#### 3.4.3. Microbiological Study

No bacteria (including *E. coli*), yeasts or molds were detected in 50 mL bottles stored for 365 days at 25 ± 2 °C or 5 ± 3 °C, reflecting “before-opening conditions”. In addition, no microorganisms were detected either in 250 mL bottles sampled three times a day for 35 days to simulate in-use conditions. Therefore, antimicrobial preservation was maintained during storage and during use.

### 3.5. Uniformity and Accuracy of Delivered Doses from Multidose Bottles

To evaluate the suitability of the nicardipine hydrochloride 2 mg/mL oral formulation with different combinations of syringes and bottle adapters, twenty 4 mL individual doses sampled using each device were weighed (Table 6). The test was compliant with Eur.Ph. for the four devices tested since no individual masses deviated from the average mass by more than 10%.

## 4. Discussion

Despite incentive regulations including the Pediatric Regulation in the European Union and the Pediatric Research Equity Act (PREA) in the United States, the use of off-label drugs is highly common in pediatrics, with 13–69% of off-label prescriptions in the hospital setting [16]. Oral liquid formulations are recommended for children, even beyond 6 years of age, while tablets and capsules are the main dose formulation from the age of 6 [17,18,19]. For oral liquid formulations, the European Medicines Agency (EMA) recommended that the volume administered for oral liquid formulations not exceed 5 mL for children under 5 years [17]. A retrospective analysis conducted in our center highlighted that the median volume administered of the previous 2 mg/mL nicardipine hydrochloride oral solution was 4.1 mL, which is consistent with EMA guidelines.

Since oral liquid formulations, such as solutions and suspensions, are the optimal formulation for children, extemporaneously compounded liquid formulations appear as an alternative when commercial liquid formulations are unavailable [20]. The preparation of such formulations may vary greatly among hospital and community pharmacies. In an effort to standardize formulations and operating procedures, an increasing number of studies related to the development of compounded oral liquid formulations is now available. Some formulations include commercial vehicles for oral use as excipients [21,22,23,24,25]. However, the composition of commercial vehicles is not fully described by suppliers, and modifications in the nature and proportion of the components may occur, raising issues for previous stability studies conducted using these products. In addition, teams commonly face supply shortages for commercial vehicles. Another approach avoiding the use of commercial vehicles is to focus on developing formulations using a combination of simple excipients [26,27].

Nicardipine hydrochloride is poorly soluble in water. Different methods were tested to improve its solubilization, including the acidification of the medium and the addition of polysorbate 80. Polysorbate 80 is a non-ionic surfactant used in several commercial pediatric oral medicines. Adverse reactions related to polysorbate 80 reported in the literature mainly occurred after intravenous injection [28,29]. No tolerance issues or adverse events related to the previous formulation used in our center were reported after three years of use to treat hypertension. Of note, polysorbate appears in the FDA list of Generally Recognized as Safe (GRAS) excipients. Nicardipine hydrochloride and polysorbate 80 both display a bitter taste. Children are generally attracted to sweet tastes and reject drugs with bitterness, raising issues for medication adherence [30,31]. Several methods to enhance palatability were tested in the pre-formulation process, and finally the formulation contained a mixture of sodium saccharin, simple syrup and strawberry flavor. This combination masked the bitter taste and provided a pleasant smell. Simple syrup is an excellent sweetening agent but contains 65% of glucose, which seems high, in particular for a medication administered to children two or three times a day. The proportion of simple syrup was reduced, and sodium saccharin was added to keep a sweet taste. Sodium saccharin is non-calorigenic and non-cariogenic, with a high sweetening power [32].

Finally, an antimicrobial preservative was added to prevent microbial contamination in this formulation designed to be a multidose preparation. Preservatives for oral formulations include sodium benzoate, propyl- and methylparaben and potassium sorbate. Sodium benzoate and parabens are described to be responsible for hypersensitivity reactions in neonates. Sodium benzoate is contraindicated in premature infants due to a risk of kernicterus, and parabens may act on the endocrine system [33,34]. The efficacy of potassium sorbate in antimicrobial preservation is reduced when the pH of the medium is above 6 [35], which was controlled using citrate buffer in the formulation. Since antimicrobial preservation was similar with 0.2% and 0.3% potassium sorbate, 0.2% was selected in the final formulation.

The analytical method was based on HPLC and tandem mass spectrometry for detection. The use of HPLC-MS/MS provides high sensitivity and specificity for the analysis. Mass spectrometry is based on the determination of the molecular weight of molecules and their fragments using the mass/charge ratio (*m*/*z*), allowing high specificity for detection. However, due to their high sensitivity, HPLC-MS/MS analyses are prone to variations. In addition, several consecutive dilutions are necessary to avoid the saturation of the detector signal, thus increasing systematic experimental variations. The analytical assay was initially developed and validated to quantify nicardipine hydrochloride in the previous formulation. Thus, prior to this study, the absence of a matrix effect was confirmed, and a forced degradation study was performed. The conditions of forced degradation included 50:50 mixtures of nicardipine hydrochloride formulation and 0.5M hydrochloric acid (pH of the mixture: 1.04) and 50:50 mixtures of nicardipine hydrochloride formulation with 0.5 M sodium hydroxide (pH of the mixture: 12.38), both heated to 80 °C for one hour. No degradation products appeared under acidic conditions, whereas nicardipine hydrochloride was degraded under alkaline conditions. This suggested that nicardipine hydrochloride was protected from thermal degradation in acidic conditions but not in alkaline conditions. This may explain the slight decrease in nicardipine hydrochloride in bottles stored at room temperature. Since the pH of the formulation was 4.77 to 4.93 (i.e., between 1.04 and 12.38) and the storage condition was less than 80 °C, it could be hypothesized that in these “intermediary” conditions, nicardipine hydrochloride was slightly degraded. However, none of the degradation products identified under alkaline degradation were detected concurrently with the decrease in nicardipine hydrochloride. It is possible that the amount of degradation product was below the limit of detection of the mass spectrometry detector.

An accurate sampling of nicardipine hydrochloride from multidose bottles is a prerequisite to administer the correct dose. Four sampling devices were evaluated according to European Pharmacopeia, and the uniformity of mass of the delivered doses was ensured for all the devices. Among them, Luer-Lock and Luer syringes have to be used with caution with oral liquid formulations, especially in a hospital setting, because of the risk of inadvertently administering the oral medication by the intravenous route. Wrong-route medication errors are prevented by the use of syringes with specific enteral connectors (e.g., ENfit^TM^, Nutrifit^®^) [36,37]. Combining specific enteral syringes with a specific adapter on the bottle provided the best security, ergonomics and hygiene of use by avoiding direct sampling inside the bottle.

## 5. Conclusions

A compounded liquid formulation of nicardipine hydrochloride 2 mg/mL was developed to fulfill a need for medications to treat hypertension in children. The formulation displayed good palatability and appropriate viscosity and included only simple and child-friendly excipients. Physicochemical and microbiological stability was maintained for 365 days at 25 ± 2 °C and 5 ± 3 °C. Stability was also ensured for one month in “in-use conditions”. This simple formulation, which could be easily manufactured in hospital and community pharmacies, is now used in clinical practice at our center. Further studies will provide data on pharmacokinetics, acceptability and efficacy.

## Figures and Tables

**Table 1:** Patients treated with the previous nicardipine hydrochloride 2 mg/mL oral formulation.

Number of Patients	32	
Age (years) (median [IQR])	4.5 [2.8–7]	
Sex ratio (M/F)	1.7	
Patient care units	Pediatric hematology/oncology	78.1% (*n* = 25)
	Pediatric intensive care unit	9.4% (*n* = 3)
	Pediatric department	6.3% (*n* = 2)
	Pediatric surgery department	6.3% (*n* = 2)
Number of administrations per day (mean ± sd)	2.8 ± 0.3	
Dose per administration (mg) (median, [IQR])	8.2 [4–10]	

Abbreviations: IQR, interquartile range; sd, standard deviation; M, male; F, female.

**Table 2:** Tested formulations of nicardipine hydrochloride 2 mg/mL.

Formulations	Polysorbate 80	Simple Syrup	Sterile Water	Thickening Agent	Sweetening Agent	Citric Acid/Sodium Citrate	Flavor	Results
F1	1%	To 50 mL	-	-	-	-	-	Visually clear after compounding Good palatability Too high glucose content
F2	1%	50%	To 50 mL	-	-	-	-	Visually clear Bitter taste
F3	-	-	To 50 mL	CMC 0.5% or 0.1%	-	-	-	Opalescent with yellow agglomerates on the magnet bar Bitter taste
F4	-	-	To 50 mL	HPMC 0.5%	-	-	-	Opalescent after compounding but good dispersion of nicardipine hydrochloride Too viscous Bitter taste Sedimentation after several days
F5	-	-	To 50 mL	HPMC 0.25%	-	-	-	Opalescent after compounding but good dispersion of nicardipine hydrochloride Adequate apparent viscosity Bitter taste Sedimentation after several days
F6	-	-	To 50 mL	HPMC 0.25%	Sorbitol 20% or 70%	-	-	Opalescent after compounding but good dispersion of nicardipine hydrochloride Adequate apparent viscosity Bitter taste was not totally masked Sedimentation after several days
F7	-	-	To 50 mL	HPMC 0.25%	Sodium saccharin 0.075–0.1%	-	-	Opalescent after compounding but good dispersion of nicardipine hydrochloride Adequate apparent viscosity Bitter taste masked using 0.1% sodium saccharin Sedimentation after several days
F8	-	-	To 50 mL	HPMC 0.25%	Sodium saccharin 0.1%	0.1%/0.1%	-	Opalescent after compounding but good dispersion of nicardipine hydrochloride Adequate apparent viscosity Bitter taste masked Sedimentation after several days
F9	1%	-	To 50 mL	HPMC 0.25%	Sodium saccharin 0.1%	0.1%/0.1%	-	Visually clear after compounding and after several days Adequate apparent viscosity Strong unpleasant taste
F10	0.75%	-	To 50 mL	HPMC 0.25%	Sodium saccharin 0.1%	0.1%/0.1%	-	Visually clear after compounding and after several days Adequate apparent viscosity Strong unpleasant taste
F11	0.5%	-	To 50 mL	HPMC 0.25%	Sodium saccharin 0.1%	0.1%/0.1%	-	Not visually clear after compounding
F12	0.75%	-	To 50 mL	HPMC 0.25%	Sodium saccharin 0.15%	0.1%/0.1%	-	Visually clear after compounding and after several days Adequate apparent viscosity Strong unpleasant taste
F13	0.75%	-	To 50 mL	HPMC 0.25%	Sodium saccharin 0.1%	0.1%/0.1%	3 to 10 drops (strawberry and/or raspberry)	Visually clear after compounding and after several days Adequate apparent viscosity Unpleasant taste not totally masked
F14	0.75%	38%	To 50 mL	HPMC 0.25%	Sodium saccharin 0.1%	0.1%/0.1%	4 drops (strawberry)	Visually clear after compounding and after several days Adequate apparent viscosity Good palatability

Note: Formulations F2 to F14 contained 0.2% or 0.3% potassium sorbate as antimicrobial preservative. Dashes indicate no addition. Percent corresponds to weight/volume. Abbreviations: CMC, carboxymethylcellulose; HPMC, hydroxypropylmethylcellulose.

**Table 3:** Efficacy of potassium sorbate for antimicrobial preservation.

Microorganisms	Potassium Sorbate	Concentration (CFU/mL)		
		Day 0	Day 14	Day 28
*Staphylococcus aureus*	0.2%	1.3 × 10^5^	0	0
	0.3%	2.4 × 10^5^	0	0
*Escherichia coli*	0.2%	1.6 × 10^5^	0	0
	0.3%	2.3 × 10^5^	0	0
*Pseudomonas aeruginosa*	0.2%	2.0 × 10^5^	0	0
	0.3%	6.9 × 10^5^	0	0
*Candida albicans*	0.2%	1.1 × 10^5^	0	0
	0.3%	1.1 × 10^5^	0	0
*Aspergillus brasiliensis*	0.2%	0.4 × 10^5^	0	0
	0.3%	1.0 × 10^5^	0	0

Abbreviations: CFU, colony-forming unit.

**Table 4:** Composition of nicardipine hydrochloride 2 mg/mL oral formulation.

Components	Quantities
Nicardipine hydrochloride	2 g
Polysorbate 80	7.5 g
Hydroxypropylmethylcellulose	2.5 g
Potassium sorbate	2 g
Sodium saccharin	1 g
Citric acid	1 g
Sodium citrate	1 g
Simple syrup	380 g
Strawberry flavor	80 drops
Sterile water	To 1000 mL

**Table 5:** Physical parameters of nicardipine hydrochloride 2 mg/mL assessed during the stability study.

Days	Storage Temperature	pH	Osmolality (mOsm/kg)
0	25 ± 2 °C	4.84 (±0)	1165 (±40)
	5 ± 3 °C	4.84 (±0.01)	1152 (±28)
7	25 ± 2 °C	4.77 (±0)	1164 (±21)
	5 ± 3 °C	4.83 (±0.01)	1144 (±17)
14	25 ± 2 °C	4.84 (±0)	1160 (±21)
	5 ± 3 °C	4.86 (±0)	1131 (±15)
28	25 ± 2 °C	4.85 (±0.01)	1159 (±20)
	5 ± 3 °C	4.86 (±0.01)	1139 (±27)
60	25 ± 2 °C	4.88 (±0)	1145 (±42)
	5 ± 3 °C	4.90 (±0.01)	1133 (±17)
90	25 ± 2 °C	4.91 (±0.01)	1166 (±22)
	5 ± 3 °C	4.93 (±0.01)	1155 (±14)
183	25 ± 2 °C	4.84 (±0.01)	1165 (±8)
	5 ± 3 °C	4.88 (±0.01)	1156 (±10)
268	25 ± 2 °C	4.87 (±0.01)	1172 (±5)
	5 ± 3 °C	4.89 (±0)	1156 (±11)
365	25 ± 2 °C	4.89 (±0.01)	1179 (±7)
	5 ± 3 °C	4.92 (±0)	1153 (±10)

Note: Data are expressed as mean (±SD).

**Table 6:** Uniformity and accuracy of delivered doses from multidose bottles.

Individual Doses	Luer-Lock Syringe with Adapter(g)	Luer Syringe with Adapter (g)	Syringe for Oral Use without Adapter (g)	Syringe for Oral Use with Adapter (g)
1	4.38	4.41	4.57	4.43
2	4.39	4.41	4.61	4.39
3	4.40	4.40	4.59	4.44
4	4.40	4.39	4.63	4.42
5	4.41	4.41	4.62	4.46
6	4.40	4.38	4.61	4.42
7	4.40	4.40	4.59	4.41
8	4.41	4.40	4.60	4.40
9	4.41	4.40	4.58	4.42
10	4.41	4.41	4.62	4.41
11	4.39	4.40	4.61	4.40
12	4.41	4.40	4.59	4.41
13	4.40	4.39	4.58	4.44
14	4.40	4.40	4.58	4.40
15	4.41	4.40	4.64	4.41
16	4.41	4.41	4.59	4.41
17	4.39	4.39	4.66	4.42
18	4.40	4.41	4.68	4.41
19	4.40	4.41	4.73	4.39
20	4.40	4.40	4.65	4.43
Average	4.40	4.40	4.62	4.42
10% deviation	[3.96–4.84]	[3.96–4.84]	[4.15–5.08]	[3.97–4.86]
20% deviation	[3.52–5.28]	[3.52–5.28]	[3.69–5.54]	[3.53–5.30]

## Data Availability

Not applicable.
